# Immunometabolism – The Role of Branched-Chain Amino Acids

**DOI:** 10.3389/fimmu.2022.886822

**Published:** 2022-06-23

**Authors:** Berkay Yahsi, Gurcan Gunaydin

**Affiliations:** ^1^School of Medicine, Hacettepe University, Ankara, Turkey; ^2^Department of Basic Oncology, Cancer Institute, Hacettepe University, Ankara, Turkey

**Keywords:** immunometabolism, T cells, regulatory T cells (Tregs), branched-chain amino acids (BCAAs), branched-chain keto acids (BCKAs), tumor microenvironment, isoleucine

## Abstract

Immunometabolism has been the focus of extensive research over the last years, especially in terms of augmenting anti-tumor immune responses. Regulatory T cells (Tregs) are a subset of CD4^+^ T cells, which have been known for their immunosuppressive roles in various conditions including anti-tumor immune responses. Even though several studies aimed to target Tregs in the tumor microenvironment (TME), such approaches generally result in the inhibition of the Tregs non-specifically, which may cause immunopathologies such as autoimmunity. Therefore, specifically targeting the Tregs in the TME would be vital in terms of achieving a successful and specific treatment. Recently, an association between Tregs and isoleucine, which represents one type of branched-chain amino acids (BCAAs), has been demonstrated. The presence of isoleucine seems to affect majorly Tregs, rather than conventional T cells. Considering the fact that Tregs bear several distinct metabolic features in the TME, targeting their immunometabolic pathways may be a rational approach. In this Review, we provide a general overview on the potential distinct metabolic features of T cells, especially focusing on BCAAs in Tregs as well as in their subtypes.

## Introduction

**Figure 1:**
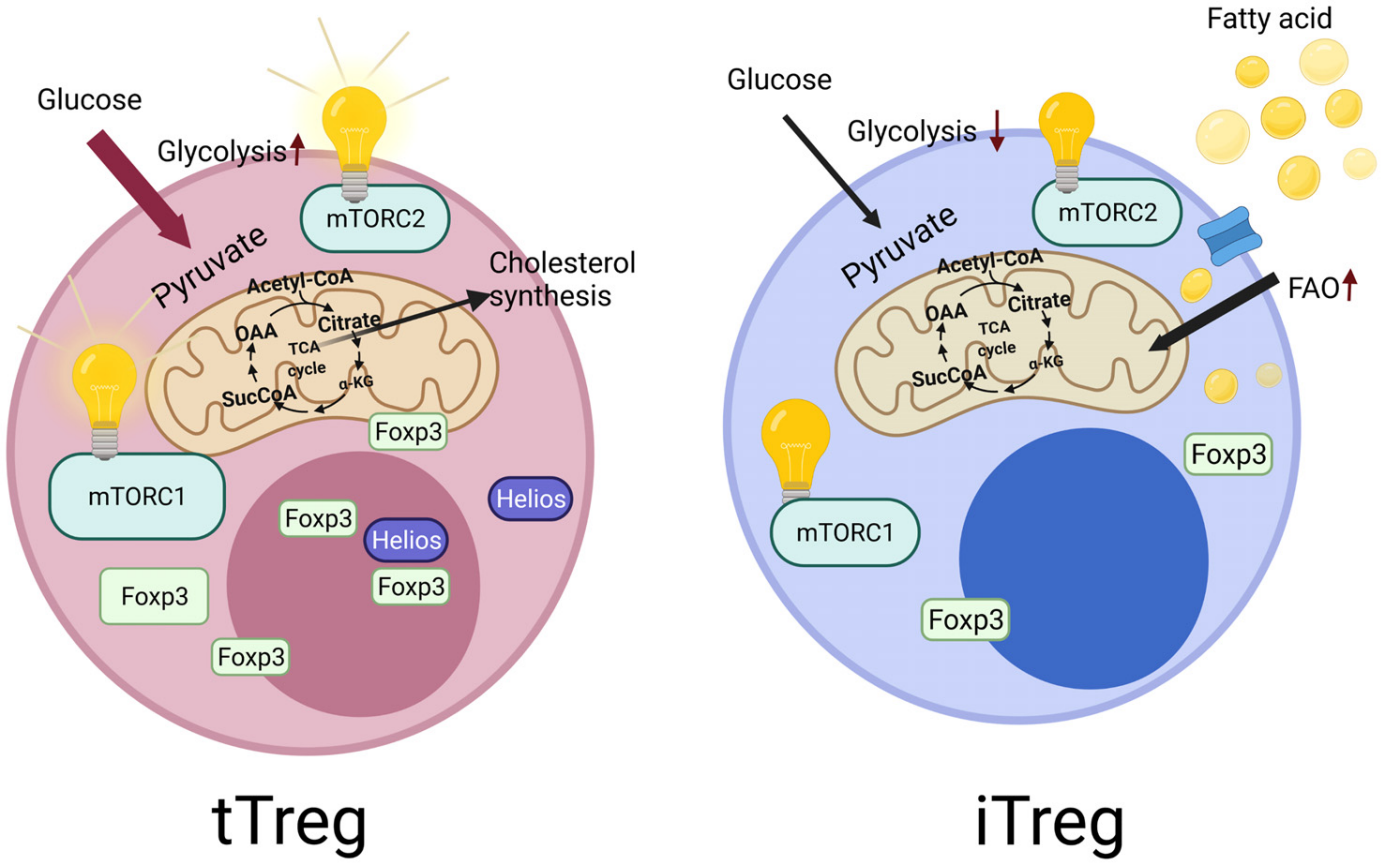
Tregs derived from the thymus called as tTregs, which constitutes the majority of Tregs in humans. They specifically express Helios, which is reported to be essential for the stability of Tregs. In contrast to tTregs, Tregs can also be generated *in vitro* from the naive CD4^+^ T cells, which are called as induced Tregs (iTregs), which do not express Helios. These two cell types also differ in terms of their metabolic regulations. iTregs mainly use fatty acid oxidation and have low levels of glycolysis. However, tTregs display higher glycolysis activity. In addition, the central metabolic regulatory pathway, mechanistic target of rapamycin (mTOR) signaling show differences in these cells. tTregs are dependent on the mechanistic target of rapamycin complex 1 and 2 (mTORC1 and mTORC2) activity for their functionality, unlike iTregs.

Regulatory T cells (Tregs) represent a specialized subpopulation of T lymphocytes that play critical roles in immune responses. They are essential for the establishment of peripheral tolerance; since autoimmune diseases and immune dysregulation are inevitable when these cells are functionally impaired (1, 2). In order for Tregs to function properly, the transcription factor forkhead box P3 (FOXP3) is critical, representing their lineage specificity and commitment (1, 2). The majority of the Tregs in humans are derived from the thymus; thus, they are called as tTreg (3, 4) (**Figure 1**). On the other hand, there also exist peripheral Tregs (pTreg), which are generated from naive CD4^+^ T cells in the periphery physiologically in healthy individuals. In addition, Tregs can be generated *in vitro* from naive CD4^+^ T cells, which are called as induced Tregs (iTregs) (5, 6). It should be noted that the expression of the Helios, which is a member of the Ikaros transcription factor family, is specific for tTreg cells (7). It was reported that Treg stability is crucially linked to the demethylated status of a conserved non-coding sequence 2 (CNS2) region, also known as Treg specific demethylation region (TSDR) in the *FOXP3* locus (8). Moreover, Helios^+^ Treg cells have been shown to have a stably demethylated TSDR compared to Helios^-^ Tregs (9). In addition, Helios is correlated with Treg stability (10). In healthy mice, most of the gut infiltrating Tregs express the retinoic acid-related orphan receptor-γt (RORγt); however, they do not express Helios, since these cells are derived from conventional T cells with the induction of an immune-suppressive milieu (11). On the other hand, another type of Treg that is of thymic origin and that expresses GATA-3 and Helios was also reported in the gut (11).

The tumor microenvironment (TME) constitutes one of the most critical factors for the elimination of the tumor as well as the effectiveness of immunotherapies. Several members of the TME (*e.g.* macrophages, fibroblasts) may be implicated in hindering anti-tumor immune responses as well as affecting the success of cancer immunotherapy (12–21). Additionally, Tregs are sometimes blamed for the failure of anti-cancer treatments such as cancer vaccines (22, 23). Moreover, Tregs highly infiltrate tumor tissues in various cancers, and their abundance is associated with poor clinical outcomes (23). As such, it was shown that the elimination of the Treg cells by anti-CD25 antibodies resulted in the eradication of the inoculated tumors in mice models (24).

Furthermore, targeting the immunometabolic pathways of Tregs may indeed be a rational approach, considering the fact that Tregs bear several distinct metabolic features in the TME (25–27). Since metabolism plays a pivotal role in biological systems, the importance of immunometabolism can be easily appreciated. In line with such findings, the metabolism of Tregs represents a vital area of investigation (28, 29). Indeed, it was proposed that Tregs have a distinct metabolic signature in several aspects, and such differences may be targeted to modulate immune responses in cancer or autoimmunity (30). Therefore, investigation of the distinct metabolic axes, as well as the potential of specifically targeting such mechanisms in T cells has been the topic of extensive research. In recent studies, the roles of amino acids for the functionality of Tregs have been better appreciated. Especially arginine and branched-chain amino acids (BCAA) are linked to the functionality of Treg cells in both *in vitro* and *in vivo* mice models through mechanistic target of rapamycin (mTOR) signaling (31, 32). In a recent study, it was demonstrated that Tregs have a different specific response to isoleucine (a BCAA) deficiency compared to conventional T cells (31). However, the underlying mechanisms of this difference remain unanswered. In this Review, we will highlight the distinct mTOR signaling and BCAA metabolism, particularly focusing on isoleucine, in T lymphocytes, aiming to propose explanations for intriguing questions. We also set out to underline the probable reasons responsible for the differences between BCAA metabolisms in Tregs and conventional T cells.

## Immunometabolism in T Cells

Immunometabolism is a relatively new and vital area of research that aims to decipher the role of metabolism in immunological reactions (33, 34). We have witnessed significant advancements in immunometabolism over the last two decades (35, 36). Indeed, lymphocytes go through various changes in terms of intracellular metabolic pathways during the activation process. Such metabolic pathways may include glycolysis, tricarboxylic acid (TCA) cycle, fatty acid oxidation, pentose phosphate pathway, fatty acid synthesis as well as amino acid metabolism (28). Indeed, naive, effector and memory T cells adapt distinct metabolic features that are associated with their differentiated states (34). Metabolic reprogramming is tightly related with T cell development, differentiation, activation as well as function. Increased requirements for ATP and biomass in order to achieve effector functions and sustain proliferation in activated T cells are met through reprogramming of the metabolism (37). It is well known that most of the adaptive and innate immune cells increase their rates of glycolysis during activation *via* aerobic glycolysis, which metabolizes glucose into lactate, because of increased biosynthetic need (38–41). For instance, effector T cells are known to be highly glycolytic while memory T cells display an oxidative metabolism. Activated T cells demonstrate high level of glucose consumption (42, 43). As such, T cells express high levels of glucose transporter 1 (Glut1) during T cell activation. This, in turn, may induce proliferation and release of cytokines (44, 45). When the T cell receptor is stimulated, costimulation *via* CD28 may increase Glut1 expression, thereby enhancing glucose uptake and glycolysis (46–48). In line with this notion, glucose deficiency can inhibit the anti-tumor effector functions of CD4^+^ T cells (49). Activated T cells were also shown to display increased oxidative phosphorylation (OXPHOS) compared to naive T cells (50).

Intriguingly, metabolic features of different subsets of T cells also display differences (51). Memory T cells are dependent on mitochondrial metabolism, whereas effector T cells utilize aerobic glycolysis (52, 53). On the contrary, Tregs do not need Glut1 or high levels of glutamine uptake (54, 55). Moreover, different effector T cell subtypes may also display differences. For instance, glutamine metabolism has distinct roles to promote Th17 while constraining Th1 and cytotoxic T lymphocyte effector cell differentiation (56, 57). Recently, a relation between glycolytic activity and epigenetic remodeling of T cells has been suggested (58–60). Moreover, Hochrein *et al.* reported that GLUT3 and ATP-citrate lyase could control the epigenetic program of Th17 cells *via* locus specific histone acetylation (61).

Analogous to naive T cells, natural killer (NK) cells were reported to preferentially utilize OXPHOS prior to activation (62, 63). Moreover, *in vitro* activated NK cells displayed induction of glycolysis and OXPHOS (62, 64), though the activation of NK cells seems to favor glycolysis, in analogy to T cells (65). Upon activation, metabolic adaptation of NK cells may be regulated by mTOR (64, 66). *in vivo* inhibition of mTOR can restrain NK proliferation early during inflammation, while mTOR inhibition after periods of inflammation may result in increased survival of innate lymphoid cell (ILC) 1 (63, 67). Similar to T cell subtypes, ILCs may be categorized into three major groups depending on the cytokines they produce, which is also analogous to the three subsets of CD4^+^ helper T cells (68). Indeed, metabolism affects the functions and development of ILCs as well as NK cells (69). In a recent study, Surace et al. demonstrated that naive ILC2s displayed higher levels of OXPHOS than NK cells (70). Furthermore, activated ILC2s were reported to rely on glycolysis and mTOR as well as continuing to fuel OXPHOS to maintain proliferation (70). Resting ILCs depend on OXPHOS, whereas activation of ILC2 may lead to increased glycolysis (71). On the other hand, activation of intestinal ILC2 in helminth infections may result in increased fatty acid oxidation - dependent OXPHOS (71).

Fatty acid oxidation may also be implicated in the regulation of the balance between effector T cells and Tregs (72). Indeed, fatty acid oxidation might promote generation of Tregs and inhibit polarization toward effector T cells (73). Interestingly, Tregs can display higher expressions of the genes related with fatty acid oxidation compared with Th17 (74). Fatty acid oxidation is also implicated in the maintenance of CD8^+^ memory T cells (75). Fessler reported that the absence of acyl-CoA carboxylase, which is a rate-limiting enzyme in fatty acid synthesis, hindered the proliferation of CD8^+^ memory T cells (76). Moreover, it was also shown that Th17 cells depended on fatty acid synthesis mediated by acyl-CoA carboxylase for their development (77).

The differences in metabolic features between various T cell subtypes suggest varying mechanisms of regulation as well as distinctive roles for metabolic alterations. As such, metabolic pathways can be pharmacologically targeted in order to achieve alterations in immune cell phenotypes. Therefore, taking advantage of the specific metabolic programs of distinct lymphocyte subtypes as a therapeutic strategy in various disease states seems to be a reasonable approach. As the tumor microenvironments is able to affect the metabolism of T cells, thereby hindering anti-tumor immune responses; treatment modalities such as checkpoint inhibition can help in attenuating the metabolic inhibition (78–81).

### mTOR Pathway and T Cells

Naive and memory cells utilize the breakdown of glucose, lipids, and amino acids metabolically to supply their ATP needs through OXPHOS (82–84). During T cell activation, T cells need increased anabolic reactions for their differentiation and function. These processes require higher energy, which is provided by the enhanced uptake of glucose and amino acids, and utilization of these molecules by glycolysis and catabolism of amino acids (especially glutaminolysis), respectively (85). Even though fatty acid oxidation is decreased during this process (85), uptake of lipids is also increased (86).

As the mTOR pathway is crucial for the regulation of most metabolic pathways (87), the role of mTOR during T cell differentiation was also well documented. The mechanistic target of rapamycin complex 1 and 2 (mTORC1 and mTORC2), in addition to mTOR protein, which has the kinase activity, also contain a scaffolding protein, regulatory-associated protein of mTOR (Raptor) (88) and rapamycin-insensitive companion of mTOR (Rictor) (89), respectively. These proteins are required for the functionality of mTORC1 and mTORC2, which are impaired by the deficiency of Raptor or Rictor, respectively. It is widely known that during T cell activation several signals can activate the mTOR pathway. There is growing of evidence that the mTOR pathway can also affect the differentiation and the fate of T cells. Indeed, two seminal articles demonstrated different aspects of the mTOR pathway in T cells (90, 91). By treating T cells with rapamycin, T cells can be induced into a state of anergy (90). In addition, it was also noted that rapamycin inhibited the differentiation of Th17, as well as promoting Treg generation (91).

Additionally, a pivotal study demonstrated that differentiations into Th1, Th2 and Th17 lineages were impaired in the absence of mTOR; and the cells differentiated into Tregs (92). In a selectively TORC1 deficient condition, differentiation into Treg lineage was not observed, indicating that TORC2 is also important for the prevention of the Treg differentiation (92). Later, it was shown that differentiation into Th1, Th17 lineages was regulated by mTORC1 signaling in a Rheb (mTORC1 activator small GTPase) dependent manner (93). Rheb deficiency impaired the generation of Th1 and Th17 responses both *in vitro* and *in vivo* (93). Interestingly, the mTORC2 signaling affected the generation of Th2 cells, unlike the deficiency of Rheb signaling (93). In parallel to such findings, Lee et al. used a conditionally Rictor deleted model, in which they observed that the absence of mTORC2 activity impaired differentiation into Th1 and Th2 lineages, but not to Th17 (94). In addition, they could show that the mTORC2 effects on Th1 cells are through Akt signaling, in contrast to its effects through PKC-θ signaling on Th2 cells (94). In contrast to the deficiency of Rheb (93), the mTORC1 deficiency significantly impaired Th2 polarization and functionality (95), underlining the essential role of mTORC1 on Th2 cells. Another study described the mTORC1 signaling kinetics in the Th17 lineage (96). They showed that the PI3K-Akt-mTORC1 axis controls Th17 differentiation by downregulation of Gfi (a negative regulator of Th17), and by the enhancement of the nuclear transport of the RORγ (96).

Moreover, Th17 differentiation is linked to the hypoxia-inducible factor (HIF1α) and deficiency of HIF1α is shown to increase the generation of Treg (97, 98). The effects of HIF1α were shown to be mTORC1 dependent (98). In a recent study, treatment of naive Tregs with rapamycin promoted differentiation of iTreg cells as Treg cell markers and FOXP3 expressions are elevated with the presence of rapamycin (99). During this induction, rapamycin decreased glycolysis, while favoring mitochondrial metabolism, and inhibition of OXPHOS suppressed FOXP3 expression, indicating the essential role of OXPHOS in iTregs (99). In parallel to this study, it was also noted that iTregs have lower levels of Glut1 expression compared to Th1, Th2 and Th17 cells. iTreg cells were reported to have lower glycolytic activity. In contrast, they had higher lipid oxidation rates and higher AMP-activated protein kinase (AMPK) activity (44). AMPK is an enzyme that plays a role in cellular energy homeostasis, functioning as a sensor of low intracellular ATP levels (100–104). When activated, AMPK phosphorylates downstream targets in order to reprogram metabolism into increased catabolism and decreased anabolism. In an asthma model, researchers demonstrated that AMPK stimulation decreased the Glut1 expression and enhanced the Treg generation (44). In addition, this phenomenon is not only obderved in iTregs but also in the differentiation of CD8^+^ memory T cells, AMPK-dependent fatty acid oxidation was shown to be essential (44, 52). This might be relevant in terms of a randomized phase 2 study in which metformin (which activates metformin) was included in the neoadjuvant therapy in postmenopausal patients, who are non-diabetic with ER^+^ breast cancer to investigate the anti-tumor effects of metformin (105).

mTORC1 may also assume roles in the cross-section between cellular and humoral immune responses. Follicular regulatory T cells differentiate from conventional Tregs and they are implicated in suppressing improper germinal center reactions *via* affecting both germinal center B cells and T follicular helper cells (106). Xu et al. utilized a model in which they genetically deleted Raptor or Rictor (essential components for mTORC1 and mTORC2, respectively [*vide supra*]). Interestingly, mTORC1 (but not mTORC2) was demonstrated to be essential for the differentiation of follicular regulatory T cells (107). mTORC1 caused phosphorylation of the transcription factor STAT3, which in turn induced the expression of the transcription factor T cell factor 1 (TCF-1), resulting in follicular regulatory T cell differentiation (107).

Most of the metabolic features of Tregs that has been explained above and in the literature are mainly focused on iTreg cells, which are generated from naive T cells in *in vitro* conditions. However, there is growing evidence that naturally occurring Tregs *in vivo* bear quite distinct metabolic regulations. As such, researchers specifically deleted the *Rptor* gene in tTreg, and it was determined that the mTORC1 signaling is crucial for Treg cell suppressive functions because CTLA4 and ICOS expression are downregulated, and Treg-cell proliferation is impaired in the absence of the mTORC1 signaling (108). Moreover, the mTORC1 was found to be responsible for the promotion of cholesterol/lipid biosynthesis whose inhibition also impaired Treg suppressive functions (108). In tTregs, this dependency on mTORC1 is similar to effector T cells, and unlike iTreg cells, as described above. Additionally, another study revealed that splenic and intratumoral Tregs could uptake more glucose than intratumoral non-Treg cells in a mouse B16 melanoma model, highlighting that *in vivo* and in the TME tTregs might display a different glucose metabolism than iTreg cells (49).

A pivotal work demonstrated that freshly isolated human *ex vivo* Tregs have higher glycolysis and oxidative phosphorylation than conventional T cells, whereas conventional T cells have higher lipid oxidation in a Seahorse assay (109). Researchers also used proteomic studies, which revealed that unstimulated *ex vivo* Tregs have higher amounts of glycolysis-related proteins, and lipid metabolism-associated proteins than conventional T cells, whereas Tregs bear lower amounts of TCA cycle proteins (109). Another study revealed that human tTregs have higher glucose and lipid metabolism than that of other T cells, and tTregs have higher Glut1 RNA levels compared to other subsets (110). In addition, tumor-derived Tregs were shown to have higher glucose and lipid metabolism, unlike effector T cells (110). When the mRNA profile of anti-CD3-activated T cells were investigated, activated Tregs have higher expression levels of glucose and lipid metabolism-related genes compared with activated effector T cell subsets (110). It is crucial to highlight the fact that in this study researchers also described the role of Toll-like receptor 8 (TLR8) in Treg metabolism. It was reported that both tumor-derived and peripheral blood Treg glucose metabolism can be inhibited with the utilization of TLR8 ligand Poly-G3. The inhibition of mTORC1–HIF1α pathway was responsible from that inhibition (110). The effectd of TLR8-mediated signaling was also evaluated in *in vivo* tumor models. Anti-tumor immune responses were enhanced with TLR8-mediated signaling by the inhibition of suppressive activity of Tregs (110).

The importance of the differences in this metabolic axis might be better appreciated given the fact that another recent study demonstrated that glucose metabolism of Tregs in peripheral blood of patients with ovarian cancer is more active than that of effector T cells of these patients (111). In addition, Tregs of patients with ovarian cancer have higher glucose metabolism than that of healthy controls and patients with benign ovarian tumors, suggesting Tregs of cancer patients might have even higher glucose metabolism than their normal state, which may be higher than effector T cells (111). Moreover, it was demonstrated that glucose metabolism and immunosuppressive functions of Tregs could be inhibited by TLR8 signaling in the SKOV3 co-culture setting (111).

It has been suggested that some amino acids could be crucial for the functions and proliferation of Tregs (31, 32). Shi et al. demonstrated that amino acids license Treg function through sustaining T cell receptor (TCR) - induced mTORC1 activity. mTORC1 activation was induced especially by arginine and leucine (32). Such findings suggested a crucial role for amino acid signaling in terms of mTORC1 activation as well as functional programming of Tregs. Therefore, we will discuss the effects of amino acids on T cell commitment and function, as well as the relationship between amino acids and the mTOR pathway in the following sections.

## Amino Acids as Signaling Molecules in T Cells

Amino acids are vital for life in a plethora of ways (112). Indeed, they can be implicated in mediating important signaling pathways in addition to their role in protein synthesis (32, 113–115). One major way of amino acid signaling is mediated by amino acid-binding proteins, including enzymes, transporters, tRNA (112). For instance, target of rapamycin was recently reported to be activated by amino acids (116). As such, Ding et al. demonstrated that amino acids could function as specific and selective signaling molecules (117). This is especially relevant for T cell signaling (*e.g.* downstream of TCR and costimulatory signals), as amino acids may influence the function (32) as well as the fate of T cells (118).

T lymphocytes that remain proximal to the antigen presenting cell (APC) - T cell interaction site after the interaction between the APCs and T cells, display a different expression of CD8, CD69, CD43, CD25, CD44 and transcription factors compared to the T lymphocytes that remain distal to the interaction during asymmetric cell division (119). Such gene expression differences suggested that the lymphocytes proximal to the interaction have an effector-like profile. In contrast, the distal lymphocytes may have a memory-like profile (119). Given this observation, researchers investigated the metabolic perspective of this asymmetric division, which led to the finding that that c-Myc was distributed asymmetrically during the process. c-Myc is a critical reprogramming transcription factor in T cells, whose deficiency impairs T cell activation (85). Importantly, c-Myc mediates transcription of amino acid transporters during T cell activation, which are needed for anabolic reactions that are indispensable for effector phenotypes (as explained above) (85). As such, the asymmetric distribution of c-Myc seemed to change the fate of these cells. Cells with high levels of c-Myc displayed effector-like phenotype, whereas cells with low levels of c-Myc displayed memory-like functions (118). Furthermore, amino acid transporter levels, amino acid content, and the activity of mTORC1 are correlated with c-Myc expression level (118).

Additionally, CD98, which is a heterodimer composed of slc3a2 and slc7a5, was shown to be expressed more on the surface of proximal daughter T cells. Moreover, the expression of CD98 was correlated with c-Myc expression levels (118). Of note, scl3a2 was found to be polarized to the contact site during T cell activation, highlighting its function in the asymmetrical division as well as in differentiation (118). Similarly, transient amino acid depletion hindered the c-Myc asymmetry, indicating that amino acids are not only crucial as simple construction units of the cells but also they may represent signaling systems that affect the decision-making processes of the cells. Indeed, amino acids can influence T cell fate in terms of deciding to become memory-like or effector-like (118). In parallel, another group showed that proximal daughter cells during asymmetrical division have higher CD8 expression and high mTORC1 activity as well as increased glycolysis (120). On the other hand, the distal daughter T cells have less mTORC1 activity and increased lipid metabolism, and long-term survival (120). Similarly, the expression of Slc7a5 mRNA is higher in CD8-high groups and CD98 protein level is enhanced in this group (120). The amino acid transport inhibitor, 2-aminobicyclo-(2,2,1)-heptane-2-carboxylic acid (BCH), inhibited the mTORC1 activation and translocation of mTOR to the lysosome during T cell activation, underlining the key role of amino acids in mTORC1 activation as well as T cell functionality (120).

Slc3a2 and slc7a5 critically regulate the uptake of BCAAs; leucine, isoleucine, and valine (121). This may further underline the possible criticial role of the BCAAs in T lymphocytes in terms of signaling systems.In a seminal work, leucine was found to be associated with induction and sustainability of TCR-mediated mTORC1 activity that is crucial for Treg suppressive modalities (32). Moreover, slc3a2-mediated uptake of BCAAs was shown to be crucial for Tregs (31). Furthermore, its expression and slc3a2 mediated isoleucine uptake were specifically augmented in Tregs compared to conventional T cells. It was demonstrated that isoleucine deficiency could hinder the proliferation and the suppressive functions of mice Tregs in *in vitro* and *in vivo* models (31). It was found that isoleucine was the most effective amino acid whose deficiency significantly inhibited Treg division and function (31). Moreover, in a seminal study, researchers demonstrated that isoleucine stimulated the mTORC1 pathway and its restriction diminished mTORC1 activity (31). However, our current knowledge does not suggest a direct protein interaction for isoleucine to activate the mTORC1 pathway, unlike leucine. It is still unclear how isoleucine can boost Treg division more than other BCAAs, and how isoleucine deficiency may significantly influence Tregs while conventional T cells seem to be less affected. Since alterations in BCAA metabolism might represent an important player for such differences, we will focus on discussing the regulation of BCAA catabolism in the next section, emphasizing how these regulations may be altered differentially among T cell subgroups.

## Catabolism of Branched-Chain Amino Acids

**Figure 2:**
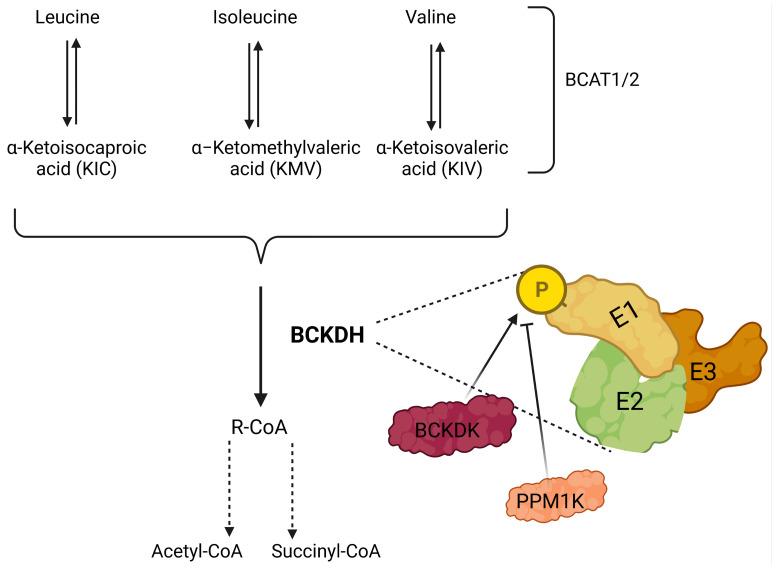
Illustration showing catabolism of branched-chain amino acids (BCAAs). BCAAs are deaminated by BCAA transaminases 1 and 2 (BCAT1/2) reversibly to form branched-chain keto acids (BCKA); leucine, isoleucine, and valine generate α-ketoisocaproate (KIC), α-keto-β-methylvalerate (KMV) and α-ketoisovalerate (KIV), respectively. BCKAs are further cleaved by the branched-chain a-keto acid dehydrogenase complex (BCKDH) to form branched-chain acyl-CoA (R-CoA) irreversibly, which may also go through several enzymatic steps to form acetyl-CoA and/or succinyl-CoA. BCKDH complex consists of three subunits E1, E2, and E3, and phosphorylation by branched-chain keto acid dehydrogenase kinase (BCKDK) inhibits the activity of BCKDH. In addition, PPM1K (protein phosphatase, Mg^2+^/Mn^2+^-dependent 1K) removes the phosphate residue and inhibits the activity of BCKDK, which results in the activation of BCKDH.

Proteinogenic BCAAs consist of leucine, isoleucine, and valine, which are essential amino acids. Since these amino acids are vital for humans, they have been extensively studied over the last decades and were investigated in terms of various aspects (122, 123). After obtaining BCAAs from the diet, cells can uptake BCAAs through transporters called, L-type amino acid transporters (LAT1-4), y^+^ L-type amino acid transporters (y^+^ LAT1-2), slc6a14, and slc6a19 (124–128). As such, it is critical to state that LAT1-2 and y^+^ LAT1-2 make an association with 4F2hc protein to form heteromeric amino acid transporters. 4F2hc protein is encoded by slc3a2 and makes the heavy subunit of this complex (121). The entry of BCAAs into the cells permits them to be processed and renders leucine, isoleucine and valine to become α-ketoisocaproate (KIC), α-keto-β-methylvalerate (KMV), and α-ketoisovalerate (KIV), respectively. This deamination reaction converts BCAAs into the keto- forms, which are collectively called branched-chain keto acids (BCKAs). The enzymes responsible for this transamination reaction are the branched-chain amino acid transaminases (BCATs) (**Figure 2**).

**Figure 3:**
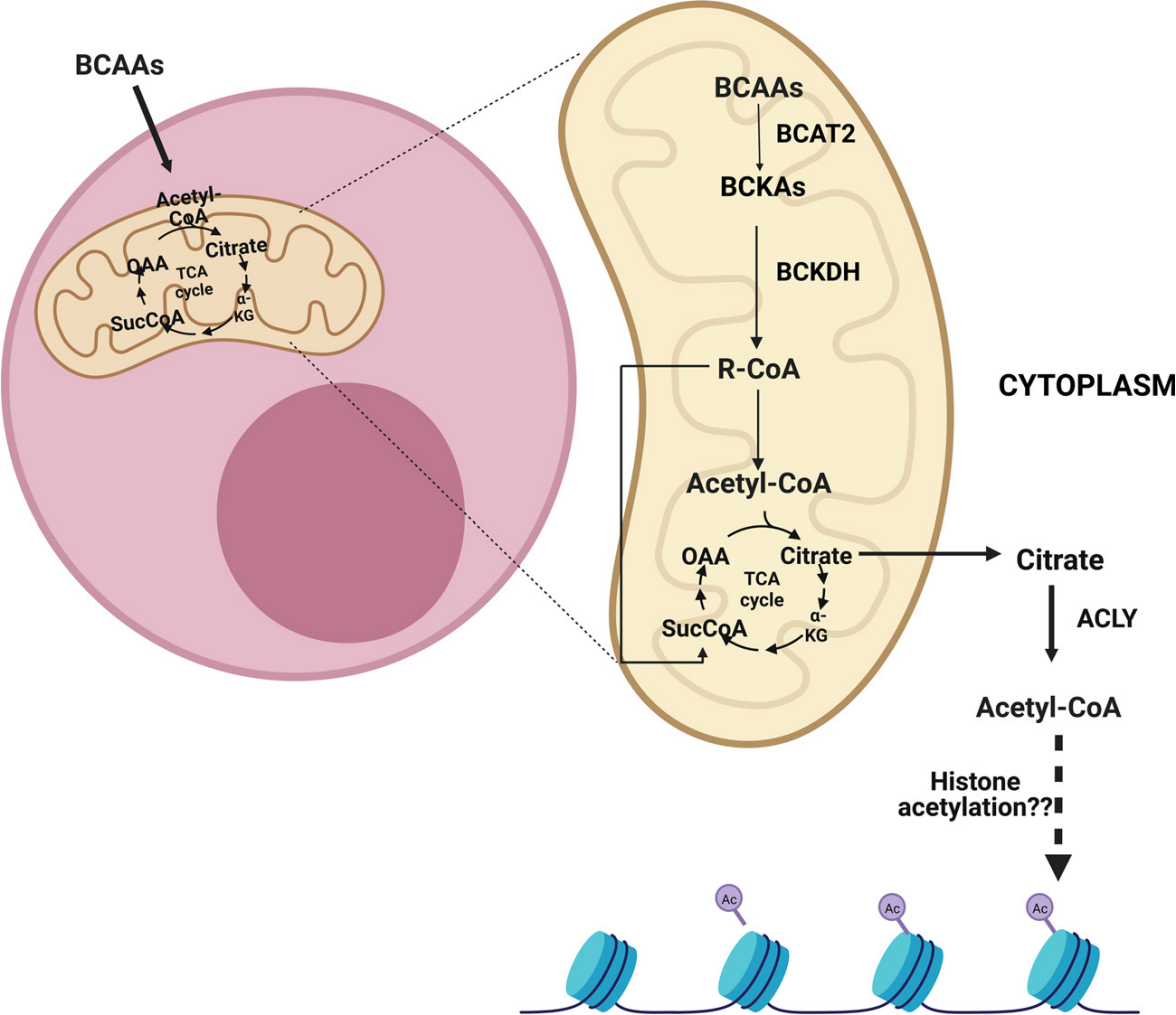
After the entry of branched-chain amino acids (BCAAs) into cells, they can be catabolized in the mitochondria into acetyl-CoA and/or succinyl-CoA depending on which BCAAs enter the reaction. Following entry into the mitochondria, BCAAs go through a transamination reaction by the branched-chain amino acid transaminase 2 (BCAT2) and become branched-chain keto acids (BCKAs). Then, the branched-chain a-keto acid dehydrogenase complex (BCKDH) forms branched-chain acyl-CoA (R-CoA) irreversibly. Subsequently, acetyl-CoA and/or succinyl-CoA can be formed as a result of several reactions. These metabolites can be transmitted to the tricarboxylic acid cycle (TCA), which also results in the formation of citrate. The citrate can be exported from mitochondria and can be converted to cytoplasmic acetyl-CoA by ATP citrate lyase (ACLY). ACLY may assume a role in sustaining cytoplasmic acetyl-CoA, which is implicated in histone acetylation.

In 1966, the first BCAT was purified from the pig heart (129). As of today, there are two forms of BCAT; *i.e.* BCAT1 is the cytoplasmic form, and BCAT2 is the mitochondrial form of this protein (**Figure 3**). Whether BCAT1 or BCAT2 carries the reaction, the result is similar, *i.e.* the amino group of the BCAA is incorporated into α-ketoglutarate to form glutamine reversibly. Even though several BCKAs can be exported from cells, some of them can be further catabolized by the BCKA dehydrogenase enzyme complex (BCKDH) which is located on the inner membrane of the mitochondria. The BCKDH highly resembles the pyruvate dehydrogenase complex. Indeed, it comprises three subunits; *i.e.* E1, E2, and E3, just like the pyruvate dehydrogenase complex. The E1 subunit is strictly regulated by the action of two proteins. The one that phosphorylates the E1 subunit is the branched-chain keto acid dehydrogenase kinase (BCKDK), which inhibits the reaction of the BCKDH. In contrast to BCKDK, the Mg^2+^/Mn^2+^-dependent 1 K protein phosphatase (PPM1K) dephosphorylates the phosphorylated form of E1 made by BCKDK, thus activating the BCKDH. After the highly regulated enzymatic activity of BCKDH, further catabolism of the BCAAs is carried out by some set-off proteins that resemble fatty acid oxidation (130). Last but not least, isoleucine is converted to acetyl-CoA and propionyl-CoA, whereas, valine and leucine are converted to propionyl-CoA and acetyl-CoA, respectively.

Several recent works highlighted the importance of the BCAA metabolism and BCKDK in cancers (131–137). It was shown that higher BCKDK expression was associated with shorter survival in colorectal cancer, and BCKDK could promote tumorigenesis *in vivo* and *ex vivo* through the mitogen-activated protein kinase (MAPK) signaling pathway (131). Additionally, a BCKDK inhibitor, phenyl butyrate, could inhibit the tumorigenesis process (131). Another study documented that increased BCKDK expression was associated with metastasis in colorectal cancer (132). In addition, genetic and pharmacological inhibition of BCKDK sensitized tumor cells to cell death in triple-negative breast cancer (TNBC) cell lines (133). Additionally, another study revealed the relation between non-small cell lung cancer (NSCLC) and BCKDK. It was observed that patients with NSCLC have higher BCAAs levels preoperatively in their blood compared to post-operative state and healthy individuals (134). Moreover, BCKDK level in NSCLC was also associated with poor prognosis and *in vitro* knockout of BCKDK decreased the proliferation of A549 and H1299 cell lines (134).

Even though these studies demonstrate the connection between BCAAs and cancer, BCAA metabolism might also have a role in the TME as it has been shown that BCKAs can induce immune-suppressive phenotypes (138). Therefore, we will discuss the relation of the BCAA catabolism with the mTOR pathway as well as the effects of this relationship on immune cells in the following sections.

## mTORC1 and Branched-Chain Amino Acids

It has been demonstrated that BCAAs are not only critical for structural purposes. They are also required for intracellular mTORC1 signaling. Cells utilize a complex system to determine current energy and nutrient availability so that they can alter their intracellular processes. Indeed, our current knowledge suggests that mTORC1 is at the center stage of this decision-making process. Through the phosphorylation of various proteins, including p70S6 kinase, eukaryotic translation initiation factor 4E binding protein 1, sterol regulatory element-binding protein (SREBP); the mTORC1 system controls the synthesis of proteins, lipids, and nucleotides (139). Detection of leucine is one of the best-understood mechanisms that cells utilize to incorporate the current nutrient status into cellular activity, particularly in the mTORC1 system. It was reported that intracellular leucine interacts with Sestrin2 and blocks the inhibitory effects of Sestrin2 on Gator2. Thus, Sestrin2 cannot inhibit Gator2, which is an activator of the mTORC1 system (113, 114). It was suggested that leucine could directly associate with a protein to activate the mTORC1 signaling system (113). Thus, Sestrin2 might represent a leucine sensor for the mTORC1 pathway (113). In addition, a second mechanism has been proposed in which leucine-derived acetyl-CoA activates mTORC1 signaling through the acetylation of Raptor protein, which is an important unit of the mTORC1 system (115). Son et al. suggested that this pathway regulated mTORC1 in a cell-type-specific manner (115). As mentioned previously, not only leucine but also isoleucine catabolism ends up with the production of acetyl-CoA with a similar enzymatic process. Furthermore, Ikeda et al. demonstrated that isoleucine is the main mTORC1 activator in Tregs (31). Therefore, the proximal part of the reactions should be considered instead of the distal part or end products, which may represent default pathways without clear regulation; if there exists a unique signal transduction in terms of BCAAs. Indeed, BCKAs seem to represent an important candidate for signal transduction since distally BCKDH has a unique modulation with various systems that causes accumulation of BCKAs in cells. Moreover, these molecules are structurally quite different from each other.

It is worth mentioning that when Newgard et al. fed mice with high fat together with BCAAs, the supplementation of BCAAs displayed increased insulin resistance, which was mTORC1-dependent since rapamycin treatment could ameliorate the BCAA-supplemented groups (140). On the other hand, Zhang et al. showed that leucine could ameliorate the insulin resistance of mice fed with a high-fat diet (141). It may be critical to state that Zhang et al. utilized only leucine, while Newgard et al. used BCAAs including isoleucine, leucine, and valine. This may suggest that isoleucine and/or valine may have distinct effects on mTORC1 or insulin resistance. Moreover, it is crucial to note that Newgard et al. highlighted the importance of the BCAA metabolism (140).

Even though insulin resistance is out of scope in our review, we believe that these differences might prove to be important in order to understand mechanistic pathways induced by different BCAAs. The different outcomes in these two studies might be linked to BCKAs. Since Biswas et al. demonstrated that excess of fatty acids attenuates the BCAA metabolism at the level of BCKDH, which results in the accumulation of the BCKAs, not BCAAs, in muscle and heart models; BCKAs might assume a vital role under such conditions (142). The researchers also measured the serum BCKA levels after 16 hours of fasting and found that serum KMV and KIC were augmented, in contrast to KIV (142). Furthermore, they analyzed the effects of treatment with different concentrations of lipids in C2C12 cells. Even though the intracellular levels of BCAAs did not change, the intracellular levels of KMV and KIV (keto- form of isoleucine and valine, respectively) were significantly increased after high dose lipid treatment, but not that of KIC (keto form of leucine). These results suggest that BCKA levels might be differently modulated (142). High-dose palmitate-treated cells also down-regulated the intermediary and distal genes of the BCAA catabolism enzymes, but proximal ones remained unchanged in C2C12 cells (142). In line with such findings, fasting for 16 hours and a high-fat & high-sucrose diet for 13 weeks, which mimics the chronic high-fat & high-sucrose conditions, caused the inhibitory phosphorylation of the BCKDH in the gastrocnemius muscle cells. Furthermore, exogenous BCKA treatment activated mTORC1 and resulted in insulin resistance. Such findings indeed confirm that augmenting the clearance of BCKAs either pharmacologically by BT-2, which is an inhibitor of BCKDK, or genetically by overexpressing the BCKDH could reverse the insulin resistance (142). Collectively, these results clearly showed that high dose lipids or fasting could restrict the BCAA catabolism at the level of BCKAs, causing the accumulation of BCKAs, which in turn results in mTORC1 signaling activation (142). Furthermore, the elevation of BCKAs during high palmitate treatment was only seen in KMV, KIV (142), which could explain how isoleucine or KMV can activate the mTORC1 system in Tregs, but not in conventional T cells if the accumulation of lipids is greater in Tregs. Therefore, the data from the insulin resistance studies suggest that high fat diet could attenuate the catabolism of BCAAs and attenuate the pathway in the BCKAs stage.

Consistent with the findings in terms of insulin resistance, the BCAA metabolism was also altered in various cancers (*vide supra*) (131–134). In a seminal article, Sivanand and Heiden reviewed the fundamental perspectives concerning the critical role of BCAA metabolism in cancer (143). Indeed, metabolism is altered in various diseases, including cancer (144, 145). Interestingly, Mayers et al. reported elevations in circulating BCAAs during pancreatic cancer progression in humans and mouse cancer models (146). Moreover, the breakdown of peripheral tissue protein in pancreas cancer might exceed the systemic requirement for BCAAs, which results in increased levels in blood (147). Moreover, production of BCKAs might be favored during disease states. Ericksen et al. showed that BCAA catabolism enzymes are lost during carcinogenesis in human hepatocellular carcinoma, except BCATs, which are elevated compared with normal liver (148). Furthermore, the expression of the BCKDK is elevated in hepatocellular carcinoma. Additionally, it was documented that a similar catabolic enzyme flux exists in various cancers, suggesting that BCATs remain normal or elevated. However, other steps of catabolic enzymes are decreased, which may result in the accumulation of BCKAs in cancers and the TME. It should also be mentioned that researchers have used KIC to test the mTORC1 activity in HepG2 cells and observed the activation of the mTORC1 signaling system. All of these studies clearly explain the physiological importance of BCKAs in various concepts. However, it remains to be explained whether accumulation of BCKAs occurs in lymphocytes and/or in the TME. It should also be clarified whether differences in lipid metabolism exist between subtypes of lymphocytes, which may be linked to accumulation of BCKAs. In the following sections, we will discuss differences in lipid metabolism and BCKAs in T cells and try to consider how these pathways might be linked to the Treg-specific isoleucine-mTORC1 activation.

## Branched-Chain Keto Acids and Immune Cells

An important point worth discussing is whether BCKAs could be generated intracellularly by CD4^+^ T cells, implicating whether BCATs can be utilized by these cells. Ananieva et al. demonstrated that the expression of BCAT is increased by the TCR signaling during the CD4^+^ T cell activation. Upon activation, the expression of the BCKDH is decreased, in contrast to the BCAT, indicating that activation may result in the accumulation of BCKAs in T cells as an intrinsic pathway (149). It should be noted that the BCAT serves as a negative regulator of the mTORC1 and decreases the metabolic activity of T cells (149). Since the increased activity of the BCAT inhibits further activation of T cells, BCAT may contribute to T cell anergy (149). The anergy is observed during the TCR signaling activation without additional co-stimulatory signals (150). Ananieva et al. also investigated the alterations in intracellular and extracellular amino acids during unstimulated, TCR stimulated with or without co-stimulation conditions for CD4^+^ T cells. They observed that all amino acids including isoleucine were significantly increased intracellularly when only TCR was stimulated (149). All amino acids except isoleucine were still significantly elevated intracellularly when TCR was stimulated together with co-stimulation; however, the level of isoleucine did not change, compared to the unstimulated condition (149). Such results seem very interesting since the increase in isoleucine levels was not observed in the condition that included co-stimulation. Further studies investigating whether isoleucine contributes to anergy may open up new avenues in terms of immune metabolism. In contrast to the general notion about CD4^+^ T cells, Tregs were demonstrated to have a high BCAT expression (151), which may also underline the importance of the BCKAs in Tregs.

**Figure 4:**
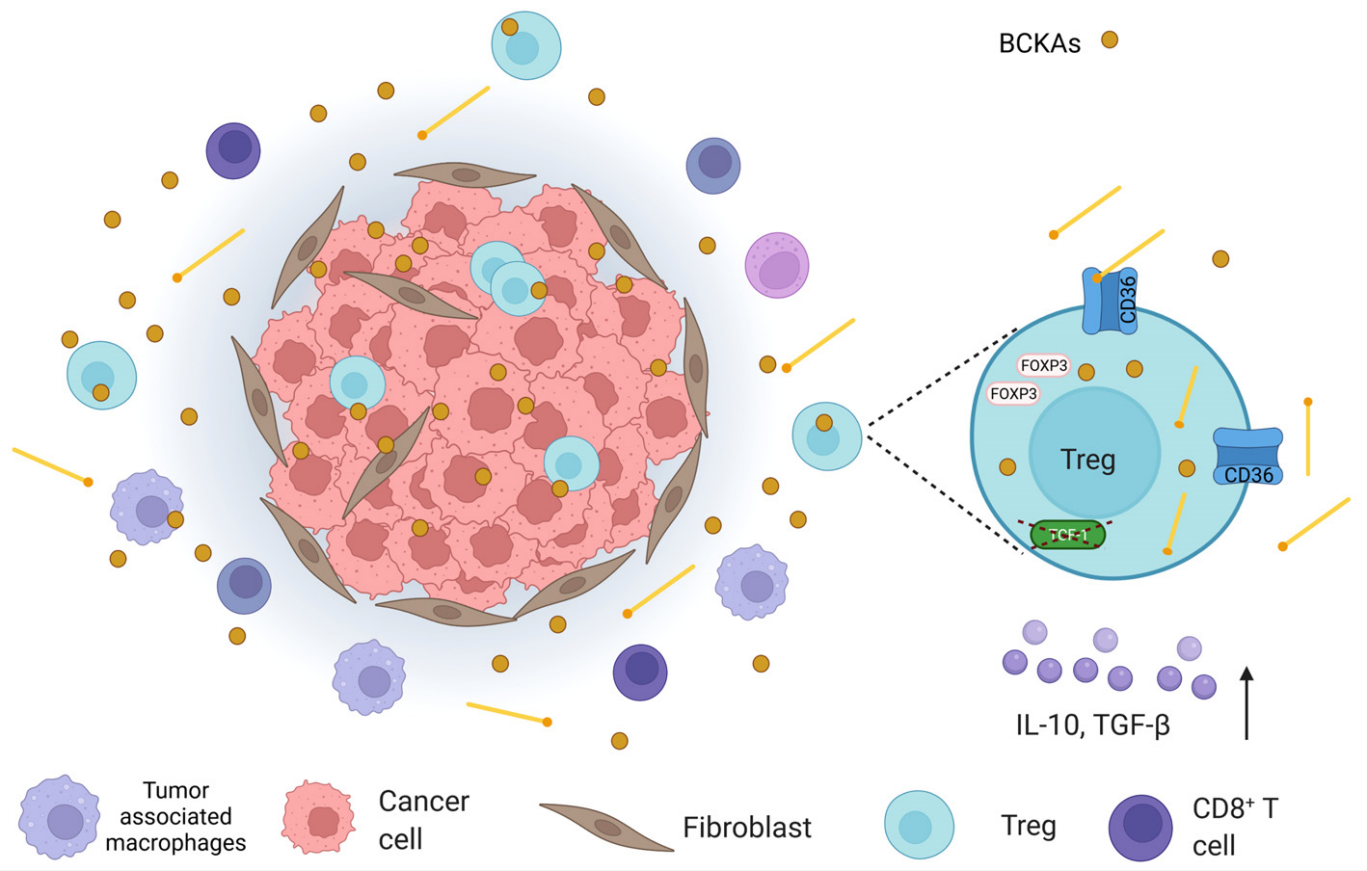
An illustration that shows the tumor microenvironment (TME) in which, apart from cancer cells, cancer-associated fibroblasts, tumor-associated macrophages, CD8^+^ T cells, and regulatory T cells (Tregs) are found. In TME, branched-chain keto acids (BCKAs), which are formed by the deamination of branched-chain amino acids (BCAAs), are present. In addition, Tregs demonstrate high levels of CD36 expression in TME. Considering the fact that CD36 mediates lipid uptake and Tregs utilize lipid oxidation as a means of energy source; they may obtain lipids from the extracellular space. High lipid uptake, in turn, can attenuate BCAA catabolism at the BCKA stage, which may also increase BCKAs in TME. Moreover, intratumoral Tregs were depicted to possess lower transcription factor T cell factor 1 (TCF-1) (in colorectal cancer). Loss of TCF-1 may contribute to both CD36 alterations and perturbation in BCAA catabolism in Tregs, which may affect their survival.

The second mechanism that may affect T cells involves acquiring BCKAs from the extracellular area. BCKAs can be released from cancer cells, as it was suggested that cancer cells could upregulate the formation of BCKAs (138, 152). The second important question that should be addressed is whether BCKAs can affect immune cells in TME, particularly the Tregs (**Figure 4**). BCKA formation from BCAAs was shown to increase in non-small cell lung cancer models and it was reported to be released back into the TME after transamination. These released BCKAs can even travel long distances as they can reach the liver to be processed (153). Researchers suggested the tumors could use BCAAs as nitrogen donors naturally *via* this process. However, it may also be important to consider that BCKAs can reshape the TME and immune cells if they can even reach to the liver. Another important study that may support this idea was conducted in a glioblastoma model (138). Silva et al. showed that the efflux of the BCKAs is mediated by monocarboxylate transporter 1 (MCT1) in glioblastoma, and the uptake of the BCKAs and the re-amination can be performed by tumor-associated macrophages (138). They also demonstrated that BCKAs could diminish the phagocytic activity of macrophages, indicating BCKAs may have an important tumor suppressor activity (138). Such an effect may favor the Tregs, while causing negative effects on conventional T cells. Such findings seem to be consistent with the effect of isoleucine-induced specific Treg expansion (31). The expansion predilection may be based on differences in BCAA catabolism between these two groups. As it was found that lipid accumulation could inhibit the BCAA catabolism except for the BCKA forming step (142), the variation may stem from the differences in lipid accumulation or processing between these groups. Therefore, differences in lipid metabolic flux seem crucial and worth discussing in the next section.

### Lymphocytes and Lipid Metabolism

The importance of fatty acids in terms of Tregs has been demonstrated clearly (154–156). Tregs were reported to have increased fatty acid oxidation and fatty acid dependency (44). Exogenous fatty acid treatment inhibited the Th1 by reducing T-bet1 expression; whereas, it favored the Treg differentiation since levels of FOXP3 increased (44). Furthermore, this phenomenon was observed in fully differentiated cells and Th1, Th2, and Th17, since the release of cytokines from these cells was diminished (44). Moreover, researchers also showed that the activation of the AMP-activated protein kinase (AMPK) and lipid metabolism favors Treg enrichment (44). Since AMPK is a major sensor of energy availability and it becomes activated during fasting (157), the BCDK activation and so the inhibitory phosphorylation on BCKDH (142) may be related to AMPK. Tregs demonstrate higher AMPK activity and this may favor their intracellular BCKAs from the BCAA differently compared to the conventional T cells.

In terms of lipid metabolism, Berod et al. highlighted the importance of the AMPK activity, which inhibits acetyl-CoA carboxylase (ACC). They proved that *de novo* fatty acid synthesis could modulate the differentiation between Treg and Th17 cell lineages (77). Th17 cells depend on *de novo* fatty acid synthesis since the blockade of the ACC favored Treg expansion but inhibited Th17 cell commitment (77). In that study, it was also shown that Tregs use exogenous lipid uptake readily, unlike Th17, which are reliant on endogenous lipid synthesis (77). It is unclear how Tregs can uptake lipids more readily. Since CD36 is one of the important mediators of lipid uptake, it might represent an important mechanism underlying such findings.

Wang et al. showed that Tregs have higher lipid metabolism activity and CD36 levels in TME than at other sites (25). Similarly, intratumoral Tregs had enhanced fatty acid uptake (25). In addition, targeting CD36 enhanced the anti-tumor activity by reducing the effects of Tregs (25). In contrast to Tregs, intratumoral CD8^+^ T cells seem to be dampened functionally when CD36 expression is increased (158). The blocking of CD36 restored their anti-tumor activity, which was responsible for inhibiting the Treg activity (158). Given such findings, Tregs may be proposed to be able to tolerate increased lipid uptake. Furthermore, they seem to increase their functionality with the elevated lipid uptake in the TME, as CD36 expression is mainly elevated intratumorally; and they lose their suppression abilities with the inhibition of CD36.

Moreover, Tregs were also demonstrated to have a different lipid metabolic axis in the TME (26). Tumor-associated Tregs were found to depend on SREBPs, which play crucial roles in lipid metabolism as transcription factors (26). In a similar perspective, inhibition of the SREBP signaling and the inhibition of the lipid synthesis could enhance the anti-tumor immunity without causing autoimmunity (26). It should be emphasized that only tumor-associated Tregs upregulated the transcription of the SREBPs in this study, and perturbing the SREBP signaling caused inhibition of tumor and enhanced the anti-tumor effects without showing autoimmune side effects (26). When the SREBP signaling inhibition was combined with immune checkpoint blockade, *e.g.* anti-PD-1, anti-tumor immune responses were further enhanced (26).. It is worth mentioning that researchers also investigated the expression of CD36 and uptake of neutral lipids in conditions of perturbed SREBP signaling, and reported that they were unaltered (26).

In parallel to such findings, another research group documented that tumor-infiltrating Tregs had increased fatty acid synthesis, which resulted in intracellular lipid accumulation (27). Even though Tregs can obtain lipids from extracellular space, they may also upregulate their lipid synthesis capacity in addition to their lipid uptake capacity (as shown at sites other than the TME), unlike Th17 cells. This would also suggest further lipid accumulation in tumor-infiltrating Tregs, which may result in further accumulation of BCKAs. In a recent study, it was shown that intratumoral Tregs have lower amounts of TCF-1 than other Tregs (compared to adjacent normal tissue and circulating T cells) (159). For this reason, we will discuss the TCF-1 and Treg relationship in the next section, as well as the potential role of TCF-1 in terms of the BCAA metabolism.

## TCF-1 and Tregs

In the classical Wnt signaling system, the transcription factor TCF-1 has a repressive function that resides on DNA segments and represses the gene expression where it is located, in case of the absence of β-catenin binding (160). However, β-catenin is stabilized and translocates to the nucleus where it binds to TCF-1, after the Wnt signal activation. In turn, they together activate the expression of specific genes that were previously repressed by TCF-1 (160).

TCF-1 is critical in terms of the development and functions of Tregs (159). The importance of TCF-1 in terms of Tregs seems to have increased after the discovery of the physical interaction between FOXP3 and TCF-1 (161). In a seminal study, van Loosdregt et al. revealed that increased Wnt signaling in Tregs reduces the suppression activity of these cells in autoimmunity models (161). Another study showed that Wnt/β-catenin signaling in Tregs resulted in the expression of the RORγt, which is a signature of Th17 cell commitment. In addition, they showed that Wnt/β-catenin signaling in Tregs not only enhances autoimmunity but also promotes colon cancer (162). WNT/β-catenin pathway has also been implicated in glucose metabolism (163). Keerthivasan et al. reported that activation of Wnt/β-catenin signaling in Tregs was associated with proinflammatory features and the promotion of colon cancer (162). In a recent study, this mechanism was further investigated. The combination of TCF-1 and FOXP3 inhibits the pro-inflammatory phenotypes in Tregs (164). When the β-catenin signaling is activated, this inhibition is perturbed, resulting in a pro-inflammatory phenotype on Tregs, which predisposes to inflammatory bowel disease and colorectal cancer (164). Another recent study reported that tumor-infiltrating Tregs demonstrated decreased TCF-1 levels in colorectal cancer (159). In addition, the researchers knocked out the TCF-1, again leading to a Th17-like phenotype but they also showed the augmented inhibition of the CD8^+^ T cells in colorectal cancer (159). Levels of FOXP3 and most of its targets were elevated, after knocking out the TCF-1 transcription factor (159). Thus, Wnt activation or TCF-1 downregulation in Tregs may be responsible for the promotion of cancers.

**Figure 5:**
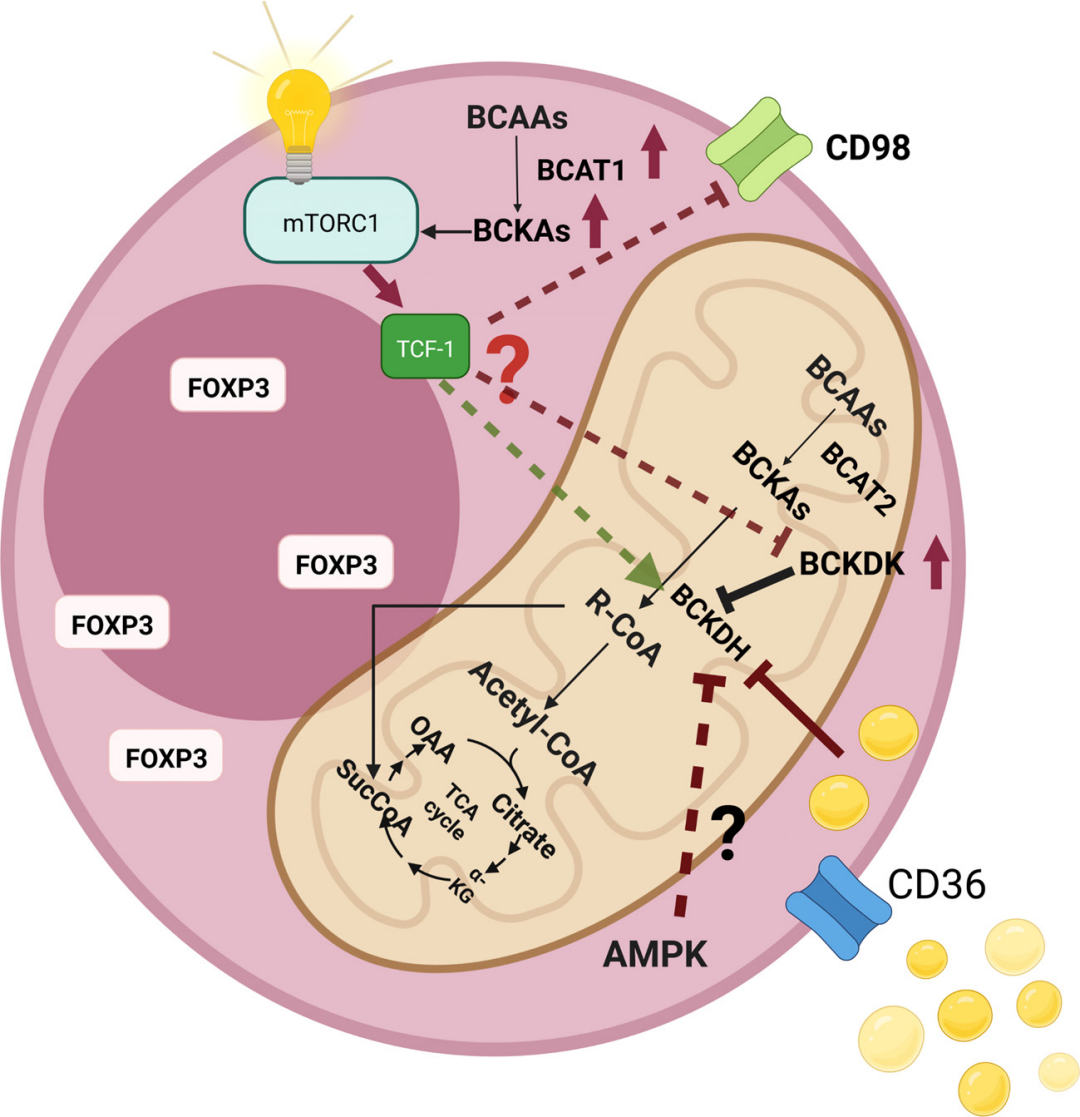
An illustration that shows the changes of branched-chain amino acid (BCAA) catabolism pathways as well as their consequences in regulatory T cells (Tregs). High levels of intracellular lipids can inhibit branched-chain a-keto acid dehydrogenase complex (BCKDH), which results in the accumulation of branched-chain keto acids (BCKAs) intracellularly. In addition, BCKAs were shown to activate the mammalian target of rapamycin complex 1 (mTORC1). Elevated levels of CD36 expression may augment intracellular lipid amount, which may contribute to the inhibition of BCKDH. Fasting is another inhibitor of BCKDH. AMP-activated protein kinase (AMPK) is a major sensor of energy availability that becomes activated during fasting. Tregs demonstrate high AMPK activity, which may further increase the levels of intracellular BCKAs. The presence of low levels of the transcription factor T cell factor 1 (TCF-1) levels in intratumoral Tregs (as in the case of colorectal cancer) may also affect the BCAA metabolic axis since TCF-1 loss was shown to significantly affect Tregs.

There may exist a metabolic aspect of such a regulation. Indeed, TCF-1 was demonstrated to maintain the metabolic signatures of T follicular helper cells (165). In addition to its critical roles in T cell development in thymus, TCF-1 has recently been implicated in peripheral T cell responses to acute/chronic infections and cancer (166, 167). Wu et al. proposed that TCF-1 could contribute to the regulation of metabolism in T follicular helper cells and promote a more oxidative metabolic profile (165). Moreover, ablation of TCF-1 was reported to expose a gene set signature to drive tissue repair and lipid metabolism (168). In addition, Germar et al. reported that TCF-1 is vital to maintain basic metabolic processes in early thymic cells (169). Ikeda et al. showed that only Helios-negative intestinal Tregs were influenced by isoleucine deficiency, but not the Helios-positive intestinal counterparts (31). Most Helios-negative cells have a Th-17-like phenotype. Thus, downregulation of TCF-1 or Wnt activation can also reshape the BCAA metabolism and result in an altered metabolic axis. For instance, it was shown that Wnt signaling could augment the LAT1 (170). Moreover, Wnt activation could increase the expression of CD36 (171). On the other hand, inhibition of the Wnt signaling can decrease the expression of CD36 (172). Additionally, Osman et al. reported that the expressions of *SLC3A2* and *BCAT2* increased, whereas the expression of *BCKDHA* decreased after the TCF-1 protein was knocked out (Supplementary Table 1 by Osman et al.) (159). Such findings suggest an altered BCAA catabolism that causes the accumulation of the BCKAs (**Figure 5**).

Importantly, IGN523, a humanized anti-CD98 (anti-SLC3A2) monoclonal antibody has been tested for relapsed or refractory acute myeloid leukemia, and preclinical data showed effective anti-tumor activity in xenograft models (173). On the other hand, LAT-1 inhibitor JPH203 showed promising results in a phase I clinical trial for biliary tract cancer (174). Even though these modalities were designed to inhibit the amino acid uptake of tumor cells, such approaches might also target Treg functions to attenuate their immunosuppressive mechanisms, which might also favor the elimination of tumor cells.

## Clinical Significance

Recent advances in the field of immunometabolism have paved the way for therapeutically targeting metabolic cellular reactions in order to control immunity and inflammation in a plethora of disease states such as cancer, autoimmune and infectious diseases (175–180). As such, metabolism of immune cells may be reprogrammed to induce or suppress inflammatory reactions. In an interesting recent study, Minhas et al. reported that rewiring myeloid glucose metabolism to restore youthful immune functions could reverse cognitive decline in aging (181). Compared to the widespread effects of conventional immunosuppressive approaches, reprogramming metabolism therapeutically has the potential to selectively target cells with specific metabolic signatures, thereby causing decreased side effects. A drug called dimethyl fumarate, which is used for the treatment of relapse-remitting multiple sclerosis, affects metabolism and regulates endogenous metabolites (35). Dimethyl fumarate is implicated in the activation of redox regulatory transcription factor Nrf2 as well as regulating pentose phosphate pathway and fatty acid metabolism.

Recently, it has been demonstrated that immune cells go through metabolic reprogramming in order to perform pro-inflammatory effector functions during viral infections such as SARS-CoV-2 (182). Therefore, metabolites with immunomodulatory properties may be used as potential therapeutic strategies in COVID-19. Itaconate, which is a key intermediate metabolite isolated from the TCA cycle, represents one of the great examples of the results of metabolic rewiring during immunity (183, 184). In addition to its metabolic roles, low plasma Itaconate levels were found to be correlated with COVID-19 disease severity (185). Furthermore, Itaconate was suggested to have a role in the regulation of type I interferons during viral infections. Though such an effect of endogenous Itaconate has not been well-established, studies with Itaconate derivatives show the possibility of such an association (184). In light of the metabolic features of and recent findings concerning Itaconate, it was proposed that Itaconate might assume a role in COVID-19 pathophysiology. As such, the potential role of Itaconate in immune responses against SARS-CoV-2 has paved the way for studies that reported synthesizing Itaconate derivatives for anti-viral screening, some of which displaying encouraging findings (182). Given these findings, it should come as no surprise to realize that Itaconate was indeed demonstrated to be antiviral against Zika virus (182, 186).

Metformin is a biguanide that is widely used in type 2 diabetes mellitus. Metformin inhibits mitochondrial electron transport chain at Complex I and activates AMPK (187). It is implicated in decreased mitochondrial reactive oxygen species (ROS) and increased fatty acid oxidation as well as inhibition of mTOR complex I (35, 188). Kelly et al. reported that metformin inhibited the production of ROS in lipopolysaccharide activated macrophages (189). Several metabolic effects of metformin are independent of AMPK, despite the fact that metformin mediated activation of AMPK leads to decreased gluconeogenic gene transcription (190). Zarrouk et al. suggested that inhibitory effects of metformin on T cells was not dependent on the expression of AMPK in T cells. They also reported that metformin could regulate proliferation of antigen activated T cells by modulating the metabolic reprogramming (191). On the other hand, metformin was reported to improve impaired B cell functions associated with type 2 diabetes mellitus (192). In a seminal study by Yin et al., normalization of CD4^+^ T cell metabolism could reverse Systemic Lupus Erythematosus (193). Moreover, metformin may inhibit BCAA derived ketoacidosis and promote metabolic homeostasis. Sonnet et al. demonstrated that metformin decreased levels of ketoisocaproic acid, which is an inhibitor of mitochondrial function (194). Metformin also restored levels of mitochondrial metabolites as well as decreasing the expression of mitochondrial BCAT (194). Epidemiological studies suggested that levels of circulating BCAAs are positively correlated with insulin resistance and Rivera et al. proposed that metformin may alter BCAA catabolism (195). They reported that metformin might suppress BCAA catabolic enzyme activity, since metformin could suppress mRNA expressions of BCAT2 and branched‐chain‐alpha‐keto acid dehydrogenase E1a (BCKDHa) protein expression of BCAT2 as well as BCKDHa activity (195). Given the findings that show BCAAs are essential for tumor growth in the setting of various biosynthetic pathways, new anti-cancer therapy strategies that target BCAA metabolism seem promising (135). Indeed, loss of BCAA catabolism was reported to display functional advantages in tumors and such an approach might be utilized *via* specific therapeutic approaches in certain tumors (148). However, it should also be taken into consideration that the function of BCAT may vary among various cancer types and the requirements of different tumors for BCAA metabolites are not homogenous (136, 196).

In addition to such well-established drugs as dimethyl fumarate and metformin in order to achieve metabolic rewiring, targeting glycolysis and/or TCA cycle with small molecules (*e.g.* targeting glycolytic enzymes, nutrient transporters) has the potential to result in desirable selective anti-inflammatory effects, given the metabolic reprogramming and enhanced aerobic glycolysis in activated immune cells (*vide supra*).

Maple syrup urine disease (MSUD; OMIM# 248600) is a disease of inborn error of metabolism, which is inherited autosomal recessively. MSUD is caused by a defect in the enzymatic activity of the BCKDH and the clinical management of MSUD is achieved by the restriction of the BCAAs. However, maintaining an adequate supply of amino acids in protein-restricted diets is critical. Indeed, a disease called Acrodermatitis dysmetabolica (AD) may develop due to isoleucine deficiency, if the restriction is performed aggressively (197–199). In the literature, it was described that AD is mainly seen because of isoleucine deficiency. As such, increasing the isoleucine dose improved clinical manifestations of AD (197–199). Currently, the exact pathogenesis underlying AD is not well known. Given the significant effects of isoleucine as well as its deficiency on Tregs (*vide supra*), future studies investigating the probable role of isoleucine deficiency on immune dysregulation, especially on the dysfunction of Tregs, in the setting of AD may prove to yield priceless information for the mechanisms underlying this poorly understood disease. Furthermore, deciphering the critical effects of amino acid metabolism on immunity might pave the way for both a better understanding of the pathogenesis of various pathological conditions and also developing of targeted therapeutic strategies.

## Concluding Remarks and Future Perspectives

In summary, several lines of evidence suggest that lipid accumulation in Tregs may arrest the BCAA catabolism at the level of BCKAs. Considering the findings of several studies, BCKAs seem to activate the mTORC1 system (the mechanism of which has not been fully understood). BCKAs tend to have varying kinetics to stimulate signaling pathways. The specific effect of isoleucine on Tregs could emerge from the keto form of isoleucine (KMV). Moreover, the arrest of BCAA catabolism might be mainly observed in Tregs, which may also explain why Tregs are affected in a different manner unlike the conventional T cells. It is worth mentioning that intratumoral Tregs demonstrate distinct metabolic axes. As in the case of colorectal cancer, it was shown that intratumoral Tregs display low expression of the TCF-1 transcription factor. Such findings may be linked to elevated levels of CD36 and altered BCAA metabolism regulation. BCAA catabolism (particularly inhibition of the BCDK), which is being currently tried in various cancer models, can have off-target effects on T cells and may inhibit the suppression of the Tregs. Therefore, the promising results that were obtained with BCDK inhibition may also be due to the dampening of the activity of Tregs, in addition to metabolic blockade of the tumor cells.

The isoleucine-induced mTORC1 activation, shown by Ikeda et al., could be based on BCAA metabolism differences between cell groups. Thus, novel therapeutic approaches can be proposed after deciphering the exact specific mechanisms. In addition, observations from MSUD patients may assist in understanding some of the unknown mechanisms, since these patients are already on a BCAA-restricted diet. Considering the fact that most studies usually focus on mice models, such approaches may also help explaining the potential biological differences in humans. Last but not least, acquiring further insight about immunometabolism might guide us in modulating the activity of Tregs in the TME *via* specifically targeting the metabolic pathways; thus, allowing for achieving increased anti-tumor immune responses.

It is well known that metabolic processes determine the function of numerous types of cells such as T cells. As such, different metabolic signatures may represent varying functional states such as development, activation and differentiation as well as memory. Transition between these states seems to require metabolic reprogramming even at the single cell level. In line with this notion, various disease states can be associated with specific metabolic profiles. Therefore, deciphering such specific signatures and discovering strategies that target or alter these metabolic profiles bear the potential for the treatment of various immunopathologies such as autoimmunity and cancer.

Even though we have witnessed significant advancements in immunometabolism over the last years, future studies are critically required in order to better understand specific metabolic processes in immune cells in various conditions as well as to achieve clinical translation. Indeed, immunometabolism provides an extra dimension to our understanding of the immune reactions in disease conditions. As such, collaborative studies of immunology, cell metabolism, biochemistry, genetics and pharmacology will play important roles in deciphering such novel mechanisms. In addition, comprehensive approaches for analysis of molecular signatures of specific cell types (*e.g.* T cells) as part of omics approaches requires bioinformatics tools. Metabolomics, which is the systematic and comprehensive analysis of all chemical processes concerning metabolites, is currently routinely applied as a tool for investigating metabolic phenomena (200, 201). Indeed, a very recent study incorporating metabolomic analyses linked epigenetic regulation of inflammatory gene expression to metabolism (61). As such, both research and therapeutic approaches that incorporate metabolomics strategies hold great promise in terms of precision medicine.

## Author Contributions

BY and GG prepared the manuscript. All authors contributed to the article and approved the submitted version.

## Conflict of Interest

The authors declare that the research was conducted in the absence of any commercial or financial relationships that could be construed as a potential conflict of interest.

## Publisher’s Note

All claims expressed in this article are solely those of the authors and do not necessarily represent those of their affiliated organizations, or those of the publisher, the editors and the reviewers. Any product that may be evaluated in this article, or claim that may be made by its manufacturer, is not guaranteed or endorsed by the publisher.

## Glossary

**Table:**

ACC	Acetyl-CoA carboxylase
ACLY	ATP citrate lyase
AD	Acrodermatitis dysmetabolica
AMPK	AMP-activated protein kinase
APC	Antigen presenting cell
BCAAs	Branched-chain amino acids
BCAT	Branched-chain amino acid transaminase
BCAT1	Branched-chain amino acid transaminase 1 (BCAT cytosolic)
BCAT2	Branched-chain amino acid transaminase 2 (BCAT mitochondrial)
BCH	2-aminobicyclo-(2,2, 1)-heptane-2-carboxylic acid
BCKAs	Branched-chain keto acids
BCKDH	BCKA dehydrogenase enzyme complex
BCKDHa	Branched-chain-alpha-keto acid dehydrogenase E1a
BCKDK	branched-chain keto acid dehydrogenase kinase
CNS2	Conserved non-coding sequence 2
FOXP3	Forkhead box P3
Glut1	Glucose transporter 1
HIF	hypoxia-inducible factor
ILC	Innate lymphoid cell
KIC	α-ketoisocaproate
KIV	α-ketoisovalerate
KMV	α-keto-β-methylvalerate
LAT	L-type amino acid transporter
MAPK	Mitogen-activated protein kinase
MCT1	Monocarboxylate transporter 1
MSUD	Maple syrup urine disease
mTOR	Mechanistic target of rapamycin
mTORC	Mechanistic target of rapamycin complex
NK	Natural killer
NSCLC	Non-small cell lung cancer
OXPHOS	Oxidative phosphorylation
PPM1K	Mg^2+^/Mn^2+^-dependent 1K protein phosphatase
Raptor	Regulatory-associated protein of mTOR
Rictor	Rapamycin-insensitive companion of mTOR
RORγt	Retinoic acid-related orphan receptor-γt
ROS	Reactive oxygen species
SREBP	Sterol regulatory element-binding protein
TCA	Tricarboxylic acid
TCF-1	T cell factor 1
TCR	T cell receptor
TLR	Toll-like receptor
TME	Tumor microenvironment
TNBC	Triple-negative breast cancer
Treg	Regulatory T cell
TSDR	Treg specific demethylation region
y^+^ LAT	y^+^ L-type amino acid transporters.
